# Cell Surface Transporters and Novel Drug Developments

**DOI:** 10.3389/fphar.2022.852938

**Published:** 2022-03-08

**Authors:** Natasha Carmichael, Philip J. R. Day

**Affiliations:** ^1^ Faculty of Biology, Medicine and Health, The University of Manchester, Manchester, United Kingdom; ^2^ School of Biological Sciences and Manchester Institute of Biotechnology, The University of Manchester, Manchester, United Kingdom; ^3^ Faculty of Health Sciences, University of Cape Town, Cape Town, South Africa

**Keywords:** transporters, therapeutic index, aptamers, chemical fragments, diffusion

## Abstract

Despite the numerous scientific and technological advances made within the last decade the attrition rates for new drug discovery remain as high as 95% for anticancer drugs. Recent drug development has been in part guided by Lipinski’s Rule of 5 (Ro5) even though many approved drugs do not comply to these rules. With Covid-19 vaccine development strategy dramatically accelerating drug development perhaps it is timely to question the generic drug development process itself to find a more efficient, cost effective, and successful approach. It is widely believed that drugs permeate cells via two methods: phospholipid bilayer diffusion and carrier mediated transporters. However, emerging evidence suggests that carrier mediated transport may be the primary mechanism of drug uptake and not diffusion as long believed. Computational biology increasingly assists drug design to achieve desirable absorption, distribution, metabolism, elimination and toxicity (ADMET) properties. Perfecting drug entry into target cells as a prerequisite to intracellular drug action is a logical and compelling route and is expected to reduce drug attrition rates, particularly gaining favour amongst chronic lifelong therapeutics. Novel drug development is rapidly expanding from the utilisation of beyond the rule of five (bRo5) to pulsatile drug delivery systems and fragment based drug design. Utilising transporters as drug targets and advocating bRo5 molecules may be the solution to increasing drug specificity, reducing dosage and toxicity and thus revolutionising drug development. This review explores the development of cell surface transporter exploitation in drug development and the relationship with improved therapeutic index.

## Introduction–Drug Development

In 1997 Lipinski’s rule of 5 (Ro5) helped transform the world of drug development, stating that poor permeation/absorption is likely if a drug has more than 5 H bond donors (HBD), 10 H bond acceptors (HBA), a molecular weight (MW) greater than 500 Da or a calculated logP value (water:octanol partition) greater than 5 (Benet et al., 2016). However, 2 decades later currently employed parameters no longer fit the Ro5. There have been statistically significant increases for MW and HBA whilst HBD and cLogP have remained fairly constant (Shultz, 2019), such that 50% of current orally administered drugs do not obey Lipinski’s rule (Zhang and Wilkinson, 2007) whilst the increase in FDA approved oral drugs since 1997 is wholly due to molecules with a molecular weight (MW) greater than 500 Da. Modern day “drug like” properties have changed greatly over recent decades, and away from those originally specified in the Ro5.

Protein modulation has benefitted through advances in analytics (Tan et al., 2016) and emerging novel targets are generally beyond the scope of Ro5 drugs and demand more innovative approaches encompassing monoclonal antibodies, small molecule inducing protein-targeted chimerics (PROTACS) and nucleic acid based therapeutics. bRo5 drug development is increasing and appears in immunology, cardiology, oncology and infectious diseases (Yang et al., 2020). HIV protease inhibitors, erythronolides (antibiotics) and NS5A inhibitors (used to treat HCV) are just a few examples of bRo5 drugs that have had a significant influence on healthcare today.

A potential flaw with Lipinski’s rule is an over-arching assumption that the primary uptake of drugs is via phospholipid bilayer diffusion. Transporters account for some 2,000 genes (Sahoo et al., 2014) and are emerging as increasingly clinically relevant, and altering transport function has been proven to affect both pharmacokinetics and pharmacodynamics. It could be that the primary uptake of drugs is in fact carrier mediated and we should adapt our drug design process in accordance. Indeed, it is beneficial to target transporters that are differentially expressed and could serve as signatures of cell type. Homozygous carriers of Organic Cation Transporter 1 (OCT1) null alleles exhibited a twofold increase in systemic exposure of fenoterol (an OCT1 substrate) leading to a 1.5-fold increase in heart rate and a 3.4-fold increase in blood glucose. The area under the curve of Organic Anionic Transporter 2B1 (OATP2B1) substrates decreased almost two-fold due to grapefruit juice, an OATP2B1 inhibitor (Zamek-Gliszczynski et al., 2018). Approved drugs targeting cell membrane transport protein are numerous and include: solute carriers (Fluoxetin, Furosemide), ATPases (Omeprazol, Digitoxin), and ABC binding cassette (Ivacaftor, Repaglinide) amongst others (Viereck et al., 2017) Exploiting transporters can have potent effects on the cells of the human body. There already exists evidence that transporters play a greater role in drug uptake than originally thought. Diffusion rate is impacted by solute concentration gradient, osmosis, electrical potential difference and pressure across a cell membrane (Sahoo et al., 2014)and if diffusion was the primary method of uptake, then transporters would not have such a significant impact on drug concentration within cells (Kell and Oliver, 2014).

**FIGURE 1:**
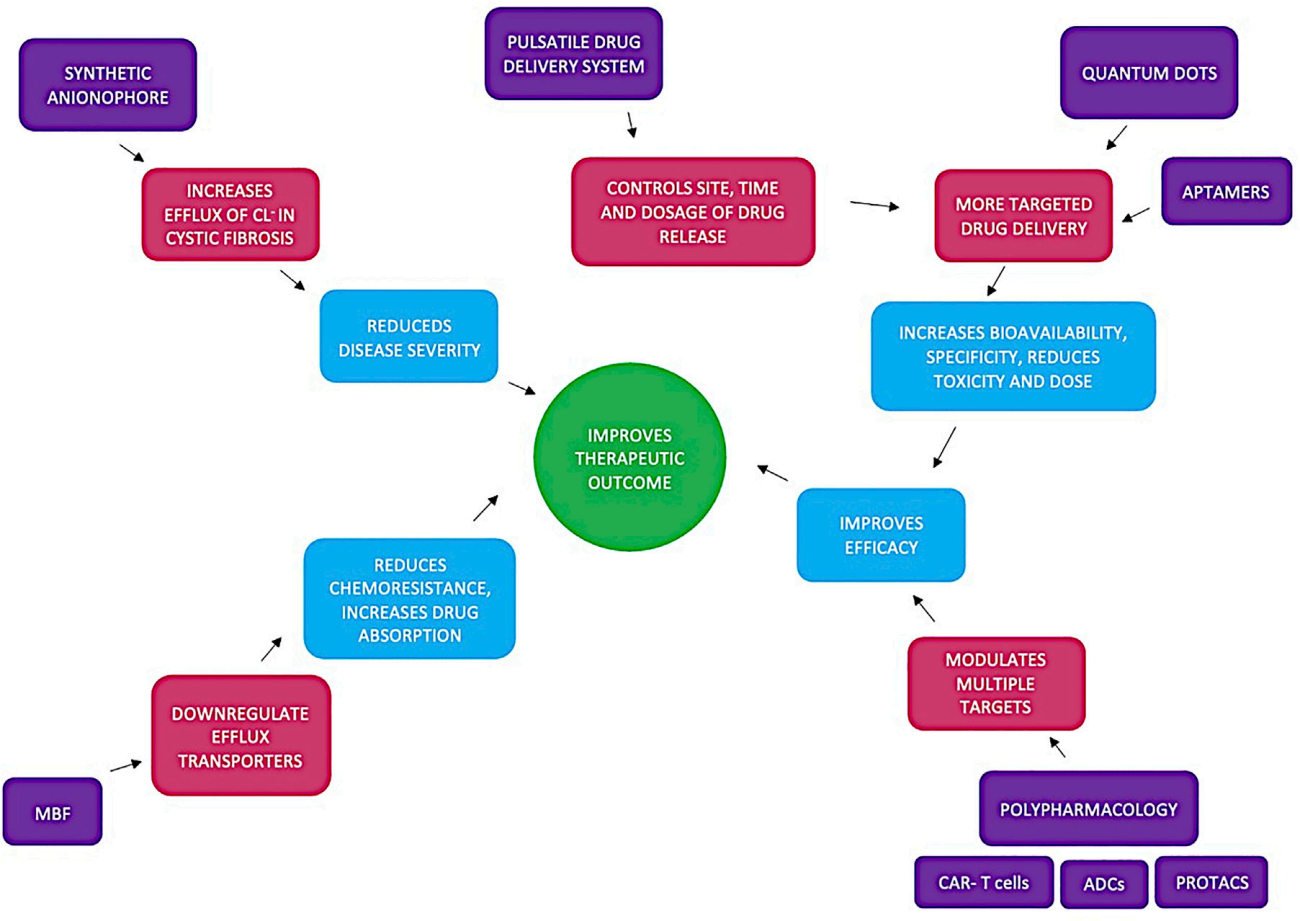
Summarises how utilising chemical fragments (such as Maybridge fragments (MBF)), polypharamcology (CAR-T cells, ADCs and protacs), quantum dots, pulsatile drug delivery systems and synthetic anionophores lead to improved therapeutic outcome. Synthetic anionophores affect ion conductivity reducing disease severity and MBF directly act on efflux transporters reducing chemoresistance and promoting drug absorption. pulsatile drug delivery systems allow for more on demand therapy whilst quantum dots promote a more targeted therapy. Both of these developments along with polypharamacology all improve efficacy leading to overall improved therapeutic outcome.

Assuming that uptake of drugs is primarily through transporters of intermediary metabolites, a more fitting rule would be “Kell’s rule of 0.5” (KRo0.5). Kell compares the similarities between marketed drugs and intermediary human metabolites using the Tanimoto index. The Molecular ACCess System keys (MACCS) fingerprint compares the chemical likeness between marketed drugs and human natural metabolites and a Tanimoto index of greater than 0.5 indicates a statistically significant similarity; 90% of marketed drugs fall within this range (O Hagan et al., 2015). In a more recent study Kell used maximum common substrate (MCS) as a further metric of comparison (O’Hagan and Kell, 2017). Despite Kell’s rule still 10% of drugs are not KRo0.5 compliant. Interaction fingerprint tools (IFT) are in development to identify drug candidates (Rácz et al., 2018). The scope of this review is presented in Figure 1 which shows some novel drug-related developments that largely exploit cell surface transporters. The review focusses on the use of small molecules and molecular fragments to improve, hasten and facilitate the successful development of drugs.

## Transporters

Transporters are membrane spanning proteins that aid the transport of ions and biomolecules across the phospholipid bilayer. Transporters can be divided into two main classes: solute carrier transporters (SLC) and ATP binding cassette (ABC) transporters. SLC transporters are involved in passive transport whereby molecules are transported across the cell membrane due to their electrochemical gradient. There are more than 300 SLC transporters found in the human body and are primarily expressed in the liver, gut, kidney and brain. They are mainly responsible for the influx of substrates such as glucose, amino acids, vitamins, neurotransmitters and inorganic ions. SLC transporters are the target of inhibitors like SSRIs for depression and sodium/glucose cotransporter inhibitors (SGLT2) for diabetes. 190 different SLC genes are associated with various loss of function Mendelian diseases (Pizzagalli et al., 2021). SLC transporters have a wide range of function. The SLC gene, SLC01B1, which encodes hepatic transporters is important in the bile elimination pathway and a polymorphism in this gene can lead to hyperbilirubinemia and jaundice (Lin et al., 2015). In presynaptic motor neurons SLC1A1-5 is responsible for the removal of glutamate from the synaptic cleft thus preventing excitotoxicity, however, in numerous neurological disorders SLC1A1-5 function/expression is reduced. Despite the need for glutamate transporter potentiators the search remains attenuated (Rives et al., 2017).

There are more than 40 ABC transporters found in humans. involved in active transport utilising the energy liberated from ATP hydrolysis and are responsible for the efflux of their substrates. They can be divided into 7 different subfamilies from ABCA to ABCG. They are abundantly expressed in the liver, intestine, kidney and brain and at least 11 are known to play key roles in multi drug resistance (MDR). These include p-gp/ABCB1, MRP1/ABCC1 and BCRP/ABCG2. P-gp, an ABC transporter, is found on the intestinal mucosal membrane, the luminal blood brain barrier, the apical membrane of hepatocytes and the epithelia of the proximal kidney tubule. P-gp transports HIV protease inhibitors, antihistamines, calcium channel blockers and anticancer drugs. Table 1 lists both the exogenous and endogenous compounds that p-gp transports. (Liu et al., 2019). Doxorubicin, a chemotherapy drug that induces immunogenic cell death (ICD) via the inhibition of topoisomerase II is ineffective against p-gp expressing cells as P-gp effluxes doxorubicin thus reducing ER stress which elicits ICD. Knockout of the gene encoding p-gp restored ICD mediated by doxorubicin highlighting the potential therapeutic importance of this transporter. The past three generations of p-gp inhibitors/modulators have not proven to be very successful due to low therapeutic index, presumably because of expression in several tissue types. However, a recent study has shown that inhibitors reducing both the amount and activity of p-gp are effective chemo-immmunesensitising agents (Kopecka et al., 2020). P-gp also has similar effects on drug concentration within the brain. A limited human study has shown that the brain to blood AUC ratio increased by 88% after administration of cyclosporine (a p-gp inhibitor) (Sasongko et al., 2005). Additionally, the knockout of the *Mdrla/Ib* gene in mice which encodes p-gp showed markedly increased levels of substrate in the brain (Schinkel et al., 1995).

**TABLE 1:** The exogenous and endogenous compounds transported by p-gp.

Transporter	Exogenous compounds	Endogenous compounds
Pgp	• Anticancer drugs (doxorubicin, daunorubicin. epirubicin. colchicine, actinomycin D. etoposide. teniposide. methotrexate, mitocycin C, paclitaxel, mitoxantrone, docetaxel, vinblastine, vincristine)	• Steroid hormones (tesoterone and aldosterone)
	• Antihypertensives (losartan. Celiorolol. reseroine. Talinolol. Nicardipine)	• Lipids (Platelet Activating Factor)
	• Antiarrhythmias (dieoxin. propafenone, auinidine. verapamil, amiodarone	• Peptides
	• Antibiotics (erythromvcin. rifampin, levofloxacin. clarithromycin, tetracycline)	• Small cytokines
	• Antivirals (amorenavir. Indinavir, nelfinair. Ritonavir, saauinavir)	
	• Antidepressants (amitrintyline. fluoxetine, paroxetine, sertraline)	
	• Immunosuppressants (cyclosporine A. sirolimus. tacrolimus, valspodar)	
	• Opioids (methadone, morphine)	
	• Statins (atorvastatin. Lovastatin)	
	• Glucocorticoids (aldosterone, cortisol, dexamethasone. Methylprednisolone)	
	• Anhistamines (fexofenadine, terfenadine)	

SLC, and ABC, transporters both play an essential role in drug uptake. Mutations and polymorphisms in genes encoding these transporters can have deleterious consequences signifying their key role in drug uptake. Therefore, drug developments could seek to utilise and manipulate these transporters to enhance therapeutic outcomes.

## Novel Drug Delivery Developments

A mutation in the CFTR gene that codes for the ABC transporter ABCC7 can cause cystic fibrosis (CF). This mutation results in a defective CFTR channel protein which leads to the dysfunction of sodium and chloride ion transport. A potential treatment for CF involves utilising synthetic anion transporters (anionophores). A fluorescence-based assay using Fischer Rat Thyroid cells identified three anionophores with promising anionic transporter activity. Further testing confirmed that the effects of these anionophores were additive to the currently used modulators, lumacaftor and ivacaftor. It is hoped that these anionophores could be developed into aerosolized drugs to improve future CF outcomes (Li et al., 2019). However, developing a synthetic transmembrane pore capable of ion conductance poses a challenge within itself. One study reports the computational design of transmembrane pores. These pores were formed by two concentric rings of alpha helices with glutamate and lysine residues incorporated to create a “polar niche”. A whole cell patch clamp test demonstrated a higher ion conductance for the transmembrane pore when compared with the control (Xu et al., 2020). Anion transmembrane transporters are being studied to selectively kill tumour cells (Soto-Cerrato et al., 2015) This nascent idea shows promising potential for the development of future synthetic ion transporters and therefore treatment of disease.

Another novel approach to drug delivery is the use of quantum dots (QDs). QDs are nanoscale semiconductor crystals which give out near infrared (NIR) emissions (>650 nm). Emissions at this region have a reduced light scattering and low tissue absorption making them highly desirable in biomedical imaging. Antibodies conjugated to QDs allowed for more efficient labelling of haematopoietic and progenitor cells when compared with fluorescently labelled antibodies (Matea et al., 2017). Quantum dots have been shown to play an important role in the plasma membrane in Alzheimer’s disease. Alzheimer’s disease is caused by the aggregation of amyloid beta (Aβ) mediated by the plasma membrane. Computer models and *in vitro* experiments have shown that graphene QDs (GQDs) disrupt the plasma membrane when binding to isomers of Aβ obstructing both beta sheet propensity and elongation (Tang et al., 2021). Graphene QDs conjugated with neuroprotective peptide glycine-proline-glutamate was shown to inhibit the aggregation of amyloid fibrils, decrease pro-inflammatory cytokine activity, increase anti-inflammatory cytokine activity as well as learning and memory capacity (Xiao et al., 2016). A molecular dynamic (MD) simulation exploring the translocation of GQDs across the phosphatidylcholine membrane found that GQDs were first adsorbed parallel to the membrane and then permeated the membrane by bending their structure. Small GQDs induced very little damage to the plasma membrane whilst larger GQDs exhibited a greater degree of damage (Kong et al., 2021).

Furthermore, QDs aid in the delivery of drugs to specific cellular targets. Controlled intracellular release of doxorubicin was achieved by ZnO pH responsive QDs. A hyaluronic acid ligand was conjugated to the ZnO QD to promote binding to CD44 (an over-expressed glycoprotein in cancerous cells). In acidic conditions the release of Zn^2+^ triggered the dissociation of the drug and thus a controlled doxorubicin release (Cai et al., 2016).

A new focus in current drug development is pulsatile drug delivery systems (PDDS). Various pathologies, like rheumatoid arthritis, follow a circadian rhythm such that the release of proinflammatory cytokines, chemokines, phagocytosis and cell migration to inflamed tissue all peak at night (Cutolo, 2019). A proof-of-concept paper explores the idea of using a PDDS remotely controlled by ultrasound waves. The gel encased drug would be activated by an acoustic device. This novel approach would allow drug delivery to be synchronised with the onset of symptoms as well as controlling the site, time and dosage of the required drug (Ciancia et al., 2020). Another application of PDDS is tissue regeneration. Growth factors (GFs) are essential in promoting cell regeneration, however, their short half-life, poor tissue penetration and off-target side effects all limit their therapeutic use. Pulsatile drug delivery is a way to overcome this hurdle as the release of GFs could be externally triggered by light, ultrasound, temperature or magnetic and electric fields. In a rat study Brain Derived Neutrophic Factor (BDNF) was loaded into a carrier microbubble, and ultrasound was applied for 5 min. When applying ultrasound at the same resonating frequency of the microbubble, microstreaming occurs (which is “the unidirectional movement of fluids along cell membranes”). The mechanical force produced promotes drug release and increasing the amplitude/frequency of the applied ultrasound wave leads to the degradation of the microbubble. After the 5-min infusion the BDNF concentration in rat brains had markedly increased along with increased myelinated axons (Cheah et al., 2021).

PDDS have gained traction in the recent years as they allow for on demand therapy as highlighted above, however, accurate delivery of the drug still poses an issue. A novel approach which overcomes this barrier is employing nanopores. Nanochannels, like ion channels, act as gates to control water and ion flow via the transitions between its inherent hydrophobic and stimuli-induced hydration state. An electrically actuated nanochannel with a high performance ion gating property was combined with a drug delivery system for the pulsatile release of penicillin G sodium and Rhodamine B. The results of this study are promising and present both an accurate and on demand drug delivery technique (Zhang et al., 2018).

The rapid growth of chemo-informatics has transformed our knowledge of drug design. Databases and tools now exist which can identify binding sites, foretell side/off target effects, drug-drug interaction (Vilar et al., 2012) and predict compound mechanism of action and similarity. This wealth of information brings a new paradigm; polypharmacology. Polypharmacology is the utilisation of a single therapeutic agent to act on multiple targets or disease pathways. A few areas of interest within this field include Proteolysis Targeting Chimeras (PROTACS), Antibody Drug Conjugates (ADCs) and chimeric antigen receptor T Cells (CAR-T) (Chaudhari et al., 2020). PROTACS work by taking advantage of the ubiquinated protein system (UPS). This causes ubiquitination and thus degradation of the protein. Philadelphia-positive (Ph+) acute lymphoblastic leukaemia requires the expression of CDK6 (a regulatory cell cycle gene). Using PROTACS to target CDK6 in mice suppressed leukaemia and spared haematopoietic progenitor cells thus reducing neutropoenia induced by other treatment (De Dominici et al., 2020). PROTACS have a poor permeability which can be countered by their high potency. Their high specificity, prolonged pharmacodynamic effect and broad applicability make them a promising development.

Antibody-drug conjugates offers minimal harm to healthy cells. Antibodies bind to specific antigens on cancer cells and deliver the cytotoxic molecules. Anti-CD20 Monoclonal antibodies (mABs) have been shown to improve the outcome of Burkitt’s Lymphoma whilst anti-CD22 mAB have been explored in refractory/relapsed childhood and adult lymphocyte leukaemia (Hoelzer, 2013). CAR-T cells are genetically designed T cells with artificial T cell receptors. The T cells receptors bind to specific antigens on tumour producing cells and induce an immunogenic response. Anti-CD19 CAR T cells have proven to be extremely efficient in B cell acute lymphoblastic leukaemia with complete remission rate up to 90% (Wang et al., 2017).

## Fragment Based Drug Discovery

A successful drug takes 12 years to develop on average and costs roughly one billion dollars (Wasko et al., 2015). Traditionally, High-throughput screening (HTS) is used to identify leads by pharmacologically testing libraries of up to millions of compounds. This costly and time consuming process is being replaced by Virtual Screening (VS). VS uses computational models including IFTs (Yu et al., 2019) to predict how a compound will interact with the target thus greatly reducing both costs and time. Fragment Based Drug Discovery (FBDD) is a low cost and potentially more rapid alternative approach to assist drug discovery that utilises small molecules to enhance drug uptake. Fragment libraries can be used to generate progressively higher affinity chemical fragments to bind the target using approaches such as closed loop evolutionary techniques (Currin et al., 2015). These selected fragments are then optimised into potent binding agents via numerous strategies; growing, scaffolding and linking. FBDD presents an attractive alternative and has already been successful in developing a number of compounds/inhibitors (Li, 2020).

Vemurafenib is the first approved fragment based drug discovery for mutant BRAF melanoma. 40% of melanoma patients possess a BRAF v600E mutation which activates the MAPK pathway resulting in excess cell proliferation and inhibited apoptosis. Clinical trials with vemurafenib exhibited a 50–80% response rate whilst median overall survival was increased from 9 to 14 months (Garbe et al., 2018). Pexidartinib, another FBDD, has been approved to treat tenosynovial giant cell tumor (TSGT) (Lamb, 2019). It is a tyrosine kinase inhibitor (TKI) selective against the colony stimulating factor (CSF-1) receptor. CSF-1 is highly expressed in solid tumours and promotes monocyte differentiation into tumour associated macrophages (TAMS) which enhance tumour growth and metastases.

The deconstruction reconstruction technique has recently caught on within FBDD. This technique breaks down the known ligand into several fragments to identify the active drug binding site of an enzyme/molecule. These fragments are then reconstructed and modified. The deconstruction of nocaine and modafinil lead to the synthesis of piperidine-based hybrid inhibitors which exhibited excellent inhibitory effects for both norepinephrine and dopamine transporters. Utilising fragments to target transporters could help ameliorate current CNS therapeutics (Chen et al., 2015). Current FBDD mainly focusses on enzyme inhibitors (Vemurafenib and pexidartinib), however, with more research this encouraging technique could be further explored to manipulate the role of transporters to improve therapeutic outcome.

## Binary Weapons Approach

A cell’s microenvironment has a key effect on transporters. Cancerous cells shift towards a glycolytic metabolism leading to a net extracellular acid production, and the drop in pH upregulates acid extruding ion transporters NBCn1 and NHE1. Polymorphisms in NBCn1 have been associated with breast cancer whilst there was decelerated tumour growth and prolonged tumour-free survival in NBCn1 knockout mice compared to wild-type (Lee et al., 2018). NHE1, on the other hand, regulates cell volume and can impact functions such as cell migration, proliferation and thus cancer development. Therefore, the upregulation of these transporters could play an important role in cancer progression. On the other hand, TASK channels 1, 2, and 3 are pH sensitive and are activated by extracellular alkalinization and thus inhibited by the acidic tumour microenvironment. TASK channels are important in cell cycle progression and are required for apoptosis in certain cells. TASK three blockers significantly reduce cell proliferation and increase apoptosis in SKOV3 and OVCAR3 cell lines suggesting that TASK channels contribute to death avoidance in cancerous cells (Andersen et al., 2014). Utilising our understanding of how a how cell microenvironment affects the portfolio of expressed transporters will only help enhance drug delivery and development.

The binary weapons approach is an exciting approach that utilises the role of transporters and their microenvironment in drug uptake. A binary weapon is a (low molecular weight) compound that has no toxicity to a cell but when mixed with a therapeutic agent greatly assists the uptake and retention of the therapeutic agent within the target cell. An example of this is demonstrated using Maybridge fragments to enhance the toxicity of gemcitabine. Gemcitabine is a nucleoside analogue of cytosine used to treat pancreatic cancer. Once transported into the cell the drug is phosphorylated and incorporated into the DNA sequence, replacing cytosine to inhibit DNA synthesis. Gemcitabine increases the expression of ABCC2 efflux transporter 12-fold which greatly reduces the drug efficacy. However, the effects of this were reversed when used in conjunction with Maybridge fragment D1 thus increasing the toxicity of gemcitabine on Panc1 cells (Grixti et al., 2017). Using small non cytotoxic molecules to enhance drug efficacy presents both a safer and cheaper alternative to current approaches and should definitely be further explored. In particular, since the approach is to facilitate the action of therapeutic agents which may have already been developed and failed approval in clinical trials for lack of specificity or intensity of action, the binary weapons approach may have high value in resuscitating failed drugs. There are high cost benefits and time savings since these failed drugs are already developed and the binary weapons themselves are serving as a type of chaperon and not directly a drug.

## Aptamers

Another small class of molecule which shows promising potential in drug development are aptamers. Aptamers are short single stranded sequences of DNA/RNA that bind to their target with a high affinity, folding into a tertiary structure. Their mechanism is very similar to that of antibodies, yet aptamers present a plethora of advantages over them. Aptamers are generally much more stable; they can withstand repeated rounds of denaturation, are stable at room temperature and have a long shelf life. Furthermore, when synthesising aptamers there is no batch to batch variation and are cheap, they are fully defined unlike antibodies, and they also possess high modifiability. Aptamers have a wide range of therapeutic uses (Zhang et al., 2019).

In human breast cancer cells the aptamer-doxorubicin conjugate was found to inhibit the proliferation of overexpressed CD44 breast cancer cells thus improving targeted therapy (Natesh et al., 2021). Doxorubicin is a chemotherapy drug, and these drugs induce lipid-lined toroidal pores in the plasma membrane. It is thought that perhaps these pores facilitate the entry of nanoparticles, like aptamers, which deliver drugs to the intracellular region (Ashrafuzzaman, 2014). Platelet-derived growth factor receptor β (PDGFR β) is highly expressed in TNBC and using PDGFR β aptamer in conjunction with the current monoclonal antibody (mAB) therapy enhanced the antiproliferative activity of anti-programmed cell death ligand 1 mAbs (Camorani et al., 2020). Aptamer gated ion channels have been proven to be effective in the detection of cancer markers. Mucin 1 (MUC1) is an important marker in breast and epithelial cancer. A gold nanofilm-anodized aluminium oxide ion channel with an aptamer encoded in the interlayers was highly sensitive in detecting MUC1. Earlier detection of cancer markers will allow for more prompt delivery of treatment and thus better therapeutic outcomes (Pan et al., 2021). Furthermore, aptamers have a potential use in non-invasive microsurgery for tumour cell destruction. Magnetic microdisks with cell binding DNA aptamers are nano/micro-sized structures. An alternating magnetic field is applied which oscillates the microdisk, disrupting the cell membrane thus inducing cell death by apoptosis. The aptamers, attached to both the disk and the target protein, extrude the target proteins from the cell membrane thus causing damage. In a murine study, gold-coated nickel magnetic microdisks triggered the apoptosis of ascites cells after just 10 min of exposure at 100 Hz AMF (Zamay et al., 2017).

## Conclusion

There are many steps involved in administering a drug to a patient, which relate to the healthcare professional themselves. These include the right patient, right medication, right route, right patient education, right documentation, right to refuse, right assessment and right evaluation. However, right dose and right frequency are determined by the drug development process. If drug development is enhanced such that drug delivery is more targeted, then both dosage and frequency can be reduced leading to overall improved therapeutic outcomes. Inhibiting efflux transporters will reduce multidrug resistance whilst potentiating influx transporters will promote ion transport. Ultimately, targeting either of these transporters will lead to a greater drug concentration in cells thus reducing off target side effects, frequency and quantity of the drug dose needed. It is important too that the impact of transporter needs to be specific to cell type and targeting transporters that are unique signatures of cell type has immense value. In this context since transporters present on the 200 or so differentiated cell types is largely unknown, it is therefore not surprising that drug developers do not exploit specific cell surface transporters. The situation is unusual in that we have outlined that carrier mediated transport is the likely primary mechanism of drug uptake, yet drugs are continued to be developed for their action and not engineered with targeted cell uptake as a primary objective. The fact that inhibiting or potentiating transporters has such significant effects on drug concentration highlights th eir vital role. Drug development was initially greatly aided by Lipinski’s Ro5, however, over time it has become increasingly evident that fewer and fewer drugs (also known as new molecular entities) (DeRuiter and Holston, 2021) meet its or FDA/EMA benefit-risk assessment requirements. The evidence of carrier mediated transport coupled with dated Ro5 drug development suggests that we should now push for novel bRo5 molecules. The novel approaches explored above– utilising synthetic transporters, nanoparticles, pulsatile drug delivery systems, FBDD and a binary weapons approach–are just the cusp of this new era of drug development that may better fit a quantitative drug risk decision analysis (Angelis and Phillips, 2021). Future drug development should focus on both honing these approaches and manipulating transporters to improve therapeutic outcomes, as transporters may prove to be the singularly most important facet uniting specific drug uptake mechanisms.
